# Disease Progression and Outcomes in Patients With Benign Prostatic Hyperplasia: Protocol for a Multicenter Retrospective Cohort Study

**DOI:** 10.2196/84965

**Published:** 2026-03-02

**Authors:** Zexuan Lv, Jiaxing Liu, Yin Lu, Xu Zhang, Fan Gao, Bingyang Guo, Hongyu Zhang, Hongzhao Li, Chao Lv

**Affiliations:** 1 Senior Department of Urology, The Third Medical Center of PLA General Hospital, 69 Yongding Road, Haidian District, Beijing, 100039, China, 86 010 669380, 86 010 6822 3575; 2 School of Medicine, Nankai University, Tianjin, China; 3 Air Force Special Medical Center, Beijing, China

**Keywords:** benign prostatic hyperplasia, BPH, Chinese patients, disease progression, clinical outcomes, multicenter retrospective cohort study

## Abstract

**Background:**

Benign prostatic hyperplasia (BPH) is highly prevalent among aging men and may lead to progressive lower urinary tract symptoms, surgical intervention, and serious outcomes such as acute urinary retention and renal impairment. In routine clinical practice, the long-term spectrum, timing, and determinants of multiple key outcome events after BPH diagnosis remain insufficiently characterized. This evidence gap is particularly relevant in China, where care pathways, follow-up patterns, and comorbidity profiles may differ from those reported in other populations. A comprehensive real-world description of long-term outcome patterns and determinants is needed to inform risk-stratified follow-up intensity and intervention timing, where current evidence remains limited.

**Objective:**

This study aims to characterize BPH progression trajectories and associated risk factors, quantify long-term risks and time-to-event profiles of major clinical outcomes, and develop and validate prognostic models using multicenter retrospective data from the People’s Liberation Army General Hospital consortium.

**Methods:**

We will establish a multicenter retrospective cohort using routinely collected clinical data from the People’s Liberation Army General Hospital consortium (the First, Third, Fourth, Fifth, Sixth, Seventh, and Eighth Medical Centers) spanning 2001 to 2021. The index date is defined as the first recorded BPH diagnosis, and we will include patients with confirmed BPH for whom long-term follow-up data are available. To approximate incident (first-diagnosis) BPH, we applied a 2-year washout period before the index date and cleaned the cohort during assembly by excluding patients with any BPH-related records within this window. Prespecified outcomes include BPH-related surgery, urinary retention, inguinal hernia, chronic kidney disease, urothelial carcinoma, and all-cause mortality. Cox proportional hazard regression will be applied for all-cause mortality, and Fine-Gray competing risk models will be used for nonmortality end points where death may act as a competing event. Model development and validation will be performed across centers, with performance assessed using discrimination, calibration, and clinical utility metrics.

**Results:**

By mid-January 2026, data collection for the multicenter retrospective dataset (2001‐2021) had been completed, and data cleaning and harmonization had commenced. As this is a retrospective electronic health record–based study, no participant recruitment is involved; the final analytic sample size will be confirmed after harmonization and cleaning. The baseline characteristic table for cohort 1 (First Medical Center) has been completed as part of data quality control. Completion of data harmonization and the prespecified analyses (including model development and validation) is anticipated by May 2026, followed by dissemination and translation to support routine clinical decision-making.

**Conclusions:**

This protocol describes a large, multicenter, real-world cohort study designed to provide robust long-term evidence on BPH progression and clinically important outcomes in China and establish interpretable and validated prognostic models to inform risk-stratified follow-up and intervention decisions.

## Introduction

Benign prostatic hyperplasia (BPH) is one of the most common urological diseases in older men, and its prevalence increases markedly with age. Epidemiological data indicate that prevalence exceeds 50% among men aged >50 years and may approach 80% in those aged >80 years [1-3]. Clinically, BPH predominantly presents with lower urinary tract symptoms (LUTS) during the storage, voiding, and terminal phases; imaging typically demonstrates prostatic enlargement, and pathological findings are characterized by glandular and stromal hyperplasia [4-7]. With disease progression, bladder outlet obstruction may worsen and lead to dysfunction of the bladder and upper urinary tract [8,9]. Notably, BPH progression is often insidious and heterogeneous: some patients experience rapid deterioration of LUTS, whereas others develop detrusor dysfunction due to long-standing obstruction, resulting in complications such as overactive bladder, urinary retention, and recurrent urinary tract infections [10-14]. These complications not only impair quality of life but also impose substantial individual and societal burdens; however, in routine clinical practice, the long-term spectrum, timing, and determinants of different outcome events following a diagnosis of BPH have not been systematically characterized, which limits refined decision-making regarding follow-up intensity and the optimal timing of intervention [15-18].

In other countries and populations, cohort studies and clinical trials have explored progression-related outcomes and associated factors in BPH and LUTS, suggesting that age, prostate volume and related indicators, baseline symptom burden, and certain metabolic comorbidities may be associated with the risks of acute urinary retention and surgical intervention [19-23]. Evidence also supports that different treatment strategies improve symptoms and, in some settings, reduce the risk of progression-related outcomes [24-26]. Nevertheless, these findings are influenced by differences in population sources, follow-up duration, and care pathways. Many studies have relatively limited follow-up, enroll patients from single centers or under restrictive eligibility criteria, and apply nonuniform outcome definitions and assessment frameworks, which complicates estimation of long-term risks and comparison across studies [27-30]. In addition, prior analyses often focus on a single outcome or short-term end points, and fewer studies have systematically evaluated multiple key outcomes within a unified long-term framework [31]. More importantly, although individualized risk prediction tools have been proposed, limitations remain in external or multicenter validation, coverage of comorbidity profiles and low-incidence outcomes, and the handling of methodological issues such as competing risks, thereby constraining reliability and generalizability for decision support in routine practice [29,32]. Therefore, real-world studies with long-term follow-up that incorporate multiple clinically important outcomes and apply rigorous methodology are needed to strengthen the evidence chain from risk factor identification to risk stratification and management decisions [29,33,34].

Overall, existing evidence on BPH remains insufficient for a systematic assessment of disease progression, prognostic determinants, and long-term clinical outcomes, and large-scale longitudinal cohorts spanning the full “progression-outcomes-prediction” continuum are scarce [19,29,35]. In China, this evidence gap is more pronounced; rapid population aging, regional variation in health care accessibility and care pathways, and differences in real-world follow-up and treatment patterns may influence outcomes and management effectiveness, creating uncertainty regarding direct extrapolation of evidence from other countries [33,36,37]. Therefore, this study aims to leverage long-term multicenter follow-up data from the People’s Liberation Army (PLA) General Hospital consortium to quantify long-term risks of key outcomes and develop and validate interpretable prediction models to inform follow-up and intervention decisions for BPH management in China.

## Methods

### Study Population and Data Source

This multicenter retrospective cohort study will use routinely collected clinical data from the PLA General Hospital consortium (the First, Third, Fourth, Fifth, Sixth, Seventh, and Eighth Medical Centers) spanning 2001 to 2021. Patients will be divided into 2 cohorts: cohort 1 (First Medical Center) and cohort 2 (the remaining medical centers). Eligible patients include those with a confirmed diagnosis of BPH and long-term follow-up, with the date of first diagnosis serving as the baseline. Patients with prostate cancer, urethral stricture, neurogenic bladder, or missing key clinical data will be excluded.

**Figure 1.**
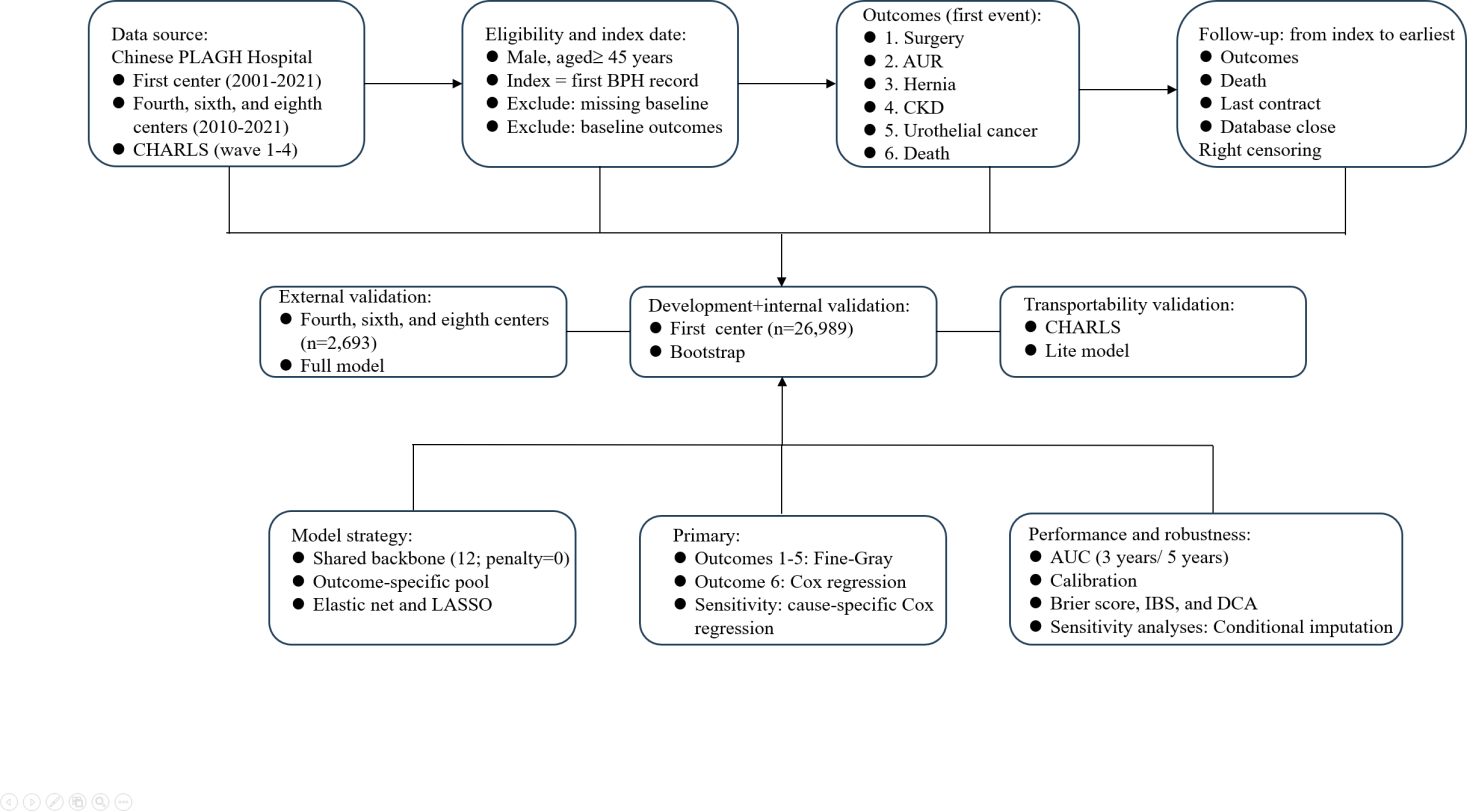
Study workflow, data sources, eligibility criteria, outcomes, follow-up, and analysis plan. The figure summarizes the data sources, inclusion and exclusion criteria and index date definition, prespecified outcomes, follow-up and censoring rules, and the overall development and validation workflow across cohorts. AUC: area under the curve; AUR: acute urinary retention; BPH: benign prostatic hyperplasia; CHARLS: China Health and Retirement Longitudinal Study; CKD: chronic kidney disease; DCA: decision curve analysis; IBS: integrated Brier score; LASSO: least absolute shrinkage and selection operator; PLAGH: People’s Liberation Army General Hospital.

Routinely collected data will be extracted from each center’s electronic health record (EHR) system, including outpatient, inpatient, and health examination records. Diagnoses and end point events will be identified using prespecified *International Classification of Diseases, 9th and 10th Revision*, codes and procedure code lists (Table 1), and relevant laboratory and clinical measurements will be retrieved from structured records. Deidentified datasets will then be transferred to the coordinating center for harmonization before analysis (Figure 1).

**Table 1.** *International Classification of Diseases, 10th Revision* (*ICD-10*), and *International Classification of Diseases, 9th Revision* (*ICD-9*), diagnostic codes for benign prostatic hyperplasia (BPH)–related comorbidities and BPH-related outcome events.^a^

	ICD-10	ICD-9
Outcomes		
Surgical treatment for BPH (any type)	Codes beginning with 0VT0, 0VT5, 0VT7, and 0VT8 (resection, incision, destruction, or enucleation of prostate via open or transurethral approach)	60.21, 60.29, 60.3‐60.5, 60.69, and 60.97
AUR^b^	R33.x	788.2
Inguinal hernia	K40.x	550.x
CKD^c^	N18.x	585.x
Urothelial carcinoma	C65, C66, and C67.x	188.x, 189.0, and 189.1
All-cause mortality	Not *ICD*^d^ based	Not *ICD* based
Baseline diseases		
BPH	N40.0 and N40.1	600.00 and 600.01
Urothelial carcinoma (renal pelvis, ureter, and bladder)	C65, C66, and C67.x	188.x, 189.0, and 189.1
Prostate cancer	C61	185
Neurogenic bladder	N31.x	344.61
Urethral stricture	N35.x	598.x
History of pelvic radiotherapy	Z92.3	V15.3
Hypertension	I10-I15	401.x
Diabetes mellitus	E10-E14	250.x
Coronary artery disease	I20-I25	410-414
Psychiatric disorders (any)	F01-F99	290-319
Insomnia (any type)	F51.0 and G47.0	780.52 and 307.42
COPD^e^	J44.x	491‐492 and 496
Asthma	J45.x	493
Chronic sinusitis	J32.x	473
Constipation	K59.0	564

a Surgical treatment was defined as any open or transurethral procedure performed for BPH, including transurethral resection, transurethral incision, laser vaporization or enucleation (eg, holmium laser enucleation of the prostate), and open simple prostatectomy. *ICD-10 Procedure Coding System* codes were identified by procedure root operations corresponding to resection, incision, destruction, or excision of the prostate (body part value: prostate). Radical prostatectomy and procedures performed for prostate malignancy (*ICD-10* C61 or *ICD-9* 185) were excluded.

b AUR: acute urinary retention.

c CKD: chronic kidney disease.

d *ICD*: *International Classification of Diseases*.

e COPD: chronic obstructive pulmonary disease.

To evaluate model transportability beyond hospital-based EHRs, we will use the China Health and Retirement Longitudinal Study (CHARLS), a nationally representative longitudinal cohort, as an independent external validation dataset. Variables and end points will be harmonized between the PLA General Hospital EHR cohort and the CHARLS using prespecified definitions; external validation will be conducted for predictors and end points that can be consistently operationalized in the CHARLS.

### Outcomes

The outcomes of this study are (1) BPH-related surgery (transurethral resection of the prostate and bipolar or laser procedures), (2) urinary retention, (3) inguinal hernia, (4) chronic kidney disease (CKD; estimated glomerular filtration rate <60 mL per minute per 1.73 m^2^ persisting for ≥90 days), (5) urothelial carcinoma, and (6) all-cause mortality (Table 1).

### Eligibility Criteria

The inclusion and exclusion criteria are outlined in Textbox 1.

Textbox 1. **Inclusion and exclusion criteria.**

**Inclusion criteria**

- Cohort construction period: January 1, 2001, to December 31, 2021.
- Benign prostatic hyperplasia (BPH) definition: BPH will be identified by either an International Prostate Symptom Score of ≥7 or a clinician-confirmed diagnosis based on digital rectal examination, imaging (ultrasound or magnetic resonance imaging), and/or pathological examination.
- Data completeness: patients must have sufficiently complete medical records for the prespecified analyses.
- Washout requirement: to approximate incident (first-diagnosis) BPH, patients must have no in-hospital BPH diagnosis record, no α-blocker or 5α-reductase inhibitor prescription, and no history of BPH-related surgery within 2 years before cohort entry.

**Exclusion criteria**

- Competing or urologic conditions: diagnosis of prostate cancer, urethral stricture, neurogenic bladder, or a history of pelvic radiotherapy before cohort entry or within 1 year after cohort entry.
- Prior outcome events: any study outcome event occurring at or before cohort entry.

### Baseline Variables for Analysis

Baseline variables used for outcome analyses and risk stratification modeling will include residence, age (years), BMI (kg/m^2^), smoking status (yes or no), alcohol consumption (yes or no), comorbidities (hypertension, diabetes mellitus, coronary artery disease, psychiatric disorders, sleep disorders, respiratory diseases, and constipation), and laboratory or clinical measures (serum creatinine, serum uric acid, triglyceride-to–high-density lipoprotein cholesterol ratio, neutrophil-to-lymphocyte ratio, alanine aminotransferase, D-dimer, total prostate-specific antigen, free prostate-specific antigen, and prostate volume).

### Data Security and Quality Assurance

Rigorous measures will be implemented to guarantee data quality, integrity, confidentiality, and statistical validity:

#### Data Quality

A multilevel verification process will be established, with clinical physicians and data managers jointly reviewing patient eligibility, baseline characteristics, and end point events. Cases not meeting criteria will be excluded to ensure authenticity.

#### Data Integrity

Baseline variables such as BMI, serum creatinine, and prostate volume will be defined as the mean values recorded at the first visit or within 6 months before or after. Missing data will be assessed using statistical methods to determine missingness patterns; only variables with low and nonbiased missing rates will be retained to avoid compromising validity.

#### Data Confidentiality

All data will be encrypted and deidentified before analysis, with personally identifiable information (eg, names and ID numbers) removed; data will be stored on secure hospital servers with access restricted to authorized team members and transferred via encrypted channels.

#### Statistical Oversight

Professional statisticians will oversee the entire process, ensuring appropriate application of Cox regression and Fine-Gray competing risk models as well as standardized procedures for Kaplan-Meier curve generation, cumulative incidence, and median time-to-event calculations. All analytic steps will be documented in a version-controlled workflow to support auditability and reproducibility.

#### Data Traceability (Audit Trail)

To ensure traceability across the workflow (data extraction, transfer, cleaning, and analysis), each dataset will be labeled with a center code and extraction time stamp, and raw files will be archived in a read-only format. All data transformations (eg, recoding, outlier handling, and missing data processing) will be recorded in a versioned data processing log, with pseudonymized unique study identifiers to track records across processing steps. Access to raw and processed datasets will be role based and logged.

### Sample Size Estimation

On the basis of Cox regression and Fine-Gray competing risk models, at least 300 to 490 events are required to analyze approximately 25 covariates [38-40]. Assuming a 10% incidence of surgery at 5 years, approximately 3334 patients will be needed. For rarer outcomes such as CKD (3% incidence), approximately 10,000 patients are required. The final sample size will be refined according to empirical event rates [40].

If, after data cleaning and cohort assembly, the effective sample size or event numbers for any prespecified end point are insufficient, we will implement the following prespecified strategies: (1) prioritization of end points with adequate events for multivariable modeling while reporting end points with sparse events descriptively and/or as exploratory analyses; (2) simplification of model specification by restricting covariates to a clinically justified core set, combining sparse categories when appropriate and using shrinkage or penalized methods to improve stability and minimize overfitting; and (3) use of alternative time-to-event approaches where needed, including penalized or stratified Cox models or time-varying effects for Cox analyses and cause-specific hazard models (or cumulative incidence estimation without multivariable regression) as alternatives when Fine-Gray models are unstable due to sparse subdistribution events. Any deviations from the initial plan and their rationale will be transparently reported.

### Statistical Analysis

To address the study objectives, analyses will comprise (1) long-term outcome profiling, (2) association analyses, and (3) prognostic risk modeling. Time-to-event methods will be used to summarize incidence, cumulative incidence, and median time to event, as appropriate. For association analyses, Cox proportional hazard regression will be applied for all-cause mortality, whereas Fine-Gray competing risk models will be used for nonmortality end points where death may act as a competing event, reporting hazard ratios or subdistribution hazard ratios with 95% CIs. Baseline variables for analysis and modeling are specified in the Baseline Variables for Analysis section. For prognostic risk modeling, multivariable prediction models will be developed and validated to support risk stratification, with performance assessed using discrimination (eg, concordance index or area under the curve), calibration, and clinical utility metrics (eg, decision curve analysis), as applicable.

Cox proportional hazard regression is appropriate for this study because the primary end points are time-to-event outcomes with right censoring and the Cox model provides an efficient and interpretable framework to account for censoring while modeling risk over follow-up. In this protocol, Cox regression is used within a prognostic (risk stratification) framework rather than solely for etiologic inference. Cox models will be applied to all-cause mortality (event 6). For nonmortality end points where death may act as a competing event, Fine-Gray competing risk models will be used to support risk estimation based on cumulative incidence.

**Figure 2.**
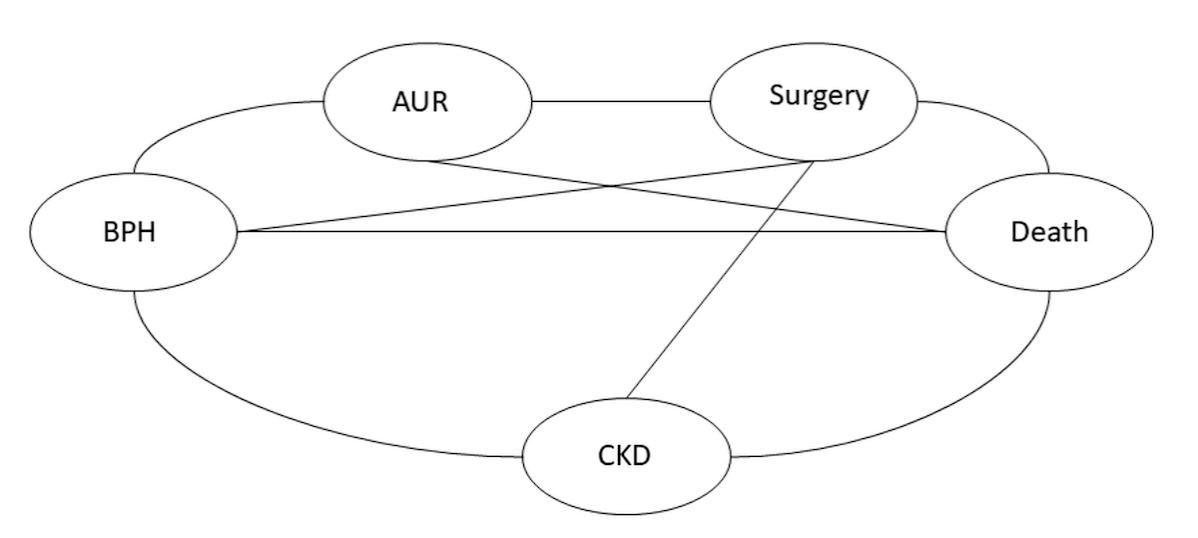
Schematic diagram of the multistate model. The final state definitions and transition pathways to be modeled will be determined based on data availability and observed event counts. AUR: acute urinary retention; BPH: benign prostatic hyperplasia; CKD: chronic kidney disease.

If supported by the empirical data structure and transition processes (eg, sufficient event counts and identifiable transition pathways), we will further explore and construct multistate models to characterize dynamic transitions between clinical states (Figure 2).

Standardized case report forms will be extracted by trained staff at each center using a unified procedure and securely transferred to the coordinating center via encrypted channels. The coordinating center will perform logic checks and randomly audit 10% of records to monitor data consistency (target inconsistency rate of ≤3%). Outliers will be identified during data cleaning and handled according to prespecified rules. Missing data will be handled using multiple imputation for all variables with missing values, and an overall sensitivity analysis will be conducted using conditional imputation. Preliminary checks indicate that most variables have <20% missingness (many with none), and all variables have <30% missingness.

Baseline characteristics will be summarized as means and SDs, medians and IQRs, or counts and percentages (Table 2).

**Table 2.** Baseline characteristic table.

Variable	Type of variable	Summary statistics
Demographic characteristics		
Residence	Categorical	Frequencies and percentages
Age (years)	Continuous	Mean and SD or median and IQR
BMI (kg/m^2^)	Continuous	Mean and SD or median and IQR
Smoking status	Binary	Frequencies and percentages
Alcohol consumption	Binary	Frequencies and percentages
Comorbidities		
Hypertension	Binary	Frequencies and percentages
Diabetes mellitus	Binary	Frequencies and percentages
Coronary artery disease	Binary	Frequencies and percentages
Psychiatric disorders	Binary	Frequencies and percentages
Sleep disorders	Binary	Frequencies and percentages
Respiratory diseases	Binary	Frequencies and percentages
Constipation	Binary	Frequencies and percentages
Laboratory measurements		
Serum creatinine (μmol/L)	Continuous	Mean and SD or median and IQR
Serum uric acid (μmol/L)	Continuous	Mean and SD or median and IQR
TG/HDL^a^	Continuous	Mean and SD or median and IQR
NLR^b^	Continuous	Mean and SD or median and IQR
ALT^c^ (U/L)	Continuous	Mean and SD or median and IQR
D-dimer (mg/L)	Continuous	Mean and SD or median and IQR
Total PSA^d^ (ng/mL)	Continuous	Mean and SD or median and IQR
Free PSA (ng/mL)	Continuous	Mean and SD or median and IQR
Prostate volume (mL)	Continuous	Mean and SD or median and IQR
Clinical outcomes		
Event 1	Categorical	Frequencies and percentages
Event 2	Categorical	Frequencies and percentages
Event 3	Categorical	Frequencies and percentages
Event 4	Categorical	Frequencies and percentages
Event 5	Categorical	Frequencies and percentages
Event 6	Categorical	Frequencies and percentages

a TG/HDL: triglyceride-to–high-density lipoprotein cholesterol ratio.

b NLR: neutrophil-to-lymphocyte ratio.

c ALT: alanine aminotransferase.

d PSA: prostate-specific antigen.

Time-to-event distributions will be summarized using appropriate survival and competing risk methods, with effect estimates from univariable and multivariable models reported as hazard ratios or subdistribution hazard ratios with 95% CIs (Multimedia Appendix 1).

Prediction models (eg, nomograms) will be constructed from multivariable results. Internal validation will use bootstrapping. External validation will include internal-external cross-validation between the 2 cohorts and an independent transportability assessment on the CHARLS (for predictors and end points that can be consistently harmonized). If miscalibration is observed in the CHARLS, we will report recalibration approaches (eg, intercept or slope updating) and present both original and recalibrated performance. Model performance will be assessed using concordance index, receiver operating characteristic curves, area under the curve, calibration plots, decision curves, and reclassification metrics (net reclassification improvement and integrated discrimination improvement).

Statistical significance will be defined as 2-sided tests, with *P*<.10 (univariate analysis) and *P*<.05 (multivariate analysis) considered significant.

### Patient and Public Involvement

As this study is a retrospective cohort analysis based on routinely collected follow-up data, patients and the public were not directly involved in formulating the research question, designing the study, or conducting the analyses. Nevertheless, we place strong emphasis on the clinical relevance and accessibility of the findings. Upon completion, results will be disseminated through peer-reviewed publications and scientific conferences and will also be shared with urology-related patient organizations and professional communities in China to facilitate translation into routine clinical decision-making and better reflect patient priorities. Building on these findings, we plan to explore prospective studies that incorporate patient and public involvement to further strengthen clinical relevance and societal impact.

### Ethical Considerations

The study protocol has been approved by the ethics committee of the PLA General Hospital (approval S2022-700-01). The entire study will be conducted in accordance with the principles of the Declaration of Helsinki and relevant ethical guidelines for medical research.

As this study is a retrospective secondary analysis of routinely collected electronic health record data, the Ethics Committee of the PLA General Hospital granted a waiver of informed consent because the analysis uses deidentified data, involves minimal risk to participants, and no direct contact with participants will occur. No participant compensation will be provided.

The study will use routinely collected clinical data for research purposes only. Access to the dataset will be restricted to authorized team members, thereby minimizing the risk of privacy breaches. Data protection measures (including encryption, deidentification, and secure storage and transfer) are described elsewhere in the Methods section.

### Dissemination and Data Availability

Findings will be disseminated through publications in peer-reviewed international and domestic journals and presented at scientific conferences, targeting clinicians, researchers, and the broader public to share key insights into the long-term prognosis of patients with BPH. Authorship will comply with the recommendations of the International Committee of Medical Journal Editors to ensure academic integrity and recognition of contributions.

Upon study completion, deidentified follow-up datasets will be archived in the Clinical Research Data Platform of the PLA General Hospital. Internal team members may conduct further subgroup analyses. External researchers may apply for access by submitting a written request detailing study aims and protocol. Access will be granted only after review and approval by the ethics committee and data platform and upon signing a data use agreement specifying scope and confidentiality obligations. Data will be retained for 20 years after study completion, after which they will be permanently destroyed according to institutional policy.

## Results

As of mid-January 2026, data collection for the multicenter retrospective dataset from the PLA General Hospital network spanning 2001 to 2021 has been completed, and data cleaning and harmonization has commenced. The baseline characteristic table for cohort 1 has been completed as part of data quality control (Table 3). In parallel, we have summarized the distributions of the core variables and outcome-specific variables, which will inform subsequent harmonization and modeling (Textbox 2). We anticipate completing data harmonization and the prespecified analyses (including model development and validation) by May 2026, followed by translation of the findings to support routine clinical decision-making.

**Table 3.** Baseline characteristic table. Continuous variables are presented as means and SDs when normally distributed and as medians and IQRs when nonnormally distributed; categorical variables are expressed as frequencies and percentages (n=26,989).

Variable	Values
Age (years), mean (SD)	61.9 (12.8)
BMI (kg/m^2^), mean (SD)	25.3 (3.5)
Smoking history, n (%)	
Yes	15,173 (56.2)
No	11,815 (43.8)
Alcohol drinking history, n (%)	
Yes	16,240 (60.2)
No	10,748 (39.8)
Hypertension, n (%)	
Yes	7757 (28.7)
No	19,232 (71.3)
Diabetes mellitus, n (%)	
Yes	4505 (16.7)
No	22,484 (83.3)
Coronary artery disease, n (%)	
Yes	5058 (18.7)
No	21,931 (81.3)
Psychiatric disorders, n (%)	
Yes	345 (1.3)
No	26,644 (98.7)
Sleep disorders, n (%)	
Yes	473 (1.8)
No	26,516 (98.2)
Respiratory diseases, n (%)	
Yes	964 (3.6)
No	26,025 (96.4)
Constipation, n (%)	
Yes	76 (0.3)
No	26,913 (99.7)
Geographic distribution, n (%)	
North China	13,726 (50.9)
Northeast China	3573 (13.2)
East China	5255 (19.5)
Central China	2683 (9.9)
South China	222 (0.8)
Southwest China	536 (2.0)
Northwest China	994 (3.7)
Serum creatinine (μmol/L), median (IQR)	79.6 (70.6-90.8)
Serum uric acid (μmol/L), median (IQR)	344.2 (293.0-400.5)
ALT^a^ (U/L), median (IQR)	20.0 (14.5-28.7)
Plasma D-dimer (mg/L), median (IQR)	0.4 (0.2-0.8)
tPSA^b^ (ng/mL), median (IQR)	1.2 (0.6-2.8)
fPSA^c^ (ng/mL), median (IQR)	0.3 (0.2-0.6)
NLR^d^, median (IQR)	0.5 (0.4-0.6)
TG/HDL-C^e^, median (IQR)	1.2 (0.8-1.7)
Prostate volume (mL), median (IQR)	31.3 (22.4-45.4)

a ALT: alanine aminotransferase.

b tPSA: total prostate-specific antigen.

c fPSA: free prostate-specific antigen.

d NLR: neutrophil-to-lymphocyte ratio.

e TG/HDL-C: triglyceride-to–high-density lipoprotein cholesterol ratio.

Textbox 2. **Configuration of core variables and outcome-specific variables across outcomes. This textbox summarizes the planned variable configuration framework for outcome analyses and model development. Core variables were initially considered for all outcomes, whereas outcome-specific variables were additionally considered for the corresponding outcome. The final variable set will be determined based on data availability, missingness, and model stability.**

**Core variables**

- Age
- BMI
- Smoking history
- Alcohol drinking history
- Hypertension
- Diabetes mellitus
- Coronary artery disease
- Psychiatric disorders
- Sleep disorders
- Respiratory diseases
- Geographic distribution
- Serum creatinine

**Outcome 1 (benign prostatic hyperplasia–related surgery)**

- Prostate volume
- Alanine aminotransferase (ALT)
- Plasma D-dimer
- Total prostate-specific antigen (tPSA)
- Free prostate-specific antigen (fPSA)
- Neutrophil-to-lymphocyte ratio (NLR)
- Triglyceride-to–high-density lipoprotein cholesterol ratio (TG/HDL-C)

**Outcome 2 (urinary retention)**

- Prostate volume
- Plasma D-dimer
- tPSA
- fPSA
- NLR
- Constipation

**Outcome 3 (inguinal hernia)**

- Prostate volume
- Constipation
- tPSA
- fPSA
- Plasma D-dimer

**Outcome 4 (chronic kidney disease)**

- Serum uric acid
- ALT
- Plasma D-dimer
- tPSA
- fPSA
- NLR
- TG/HDL-C
- Prostate volume

**Outcome 5 (urothelial carcinoma [bladder or upper tract])**

- NLR
- TG/HDL-C
- ALT
- Serum uric acid
- Constipation

**Outcome 6 (all-cause mortality)**

- NLR
- ALT
- Plasma D-dimer
- TG/HDL-C
- Serum uric acid
- Constipation

## Discussion

This study aims to establish a large-scale, long-term, multicenter retrospective cohort to systematically elucidate the full disease continuum of BPH—from progression to outcomes to prediction—and address the current knowledge gaps regarding the natural history and prognostic determinants of BPH in the Chinese population.

### Limitations

Research on the clinical course and outcomes of BPH has several limitations. First, although there is existing evidence suggesting that frailty phenotype and larger prostate volume are established risk factors for disease progression [41,42], robust quantitative data on mid- to long-term risks of key outcomes, such as surgical intervention and acute urinary retention, remain limited. Second, most studies have relatively short follow-up durations, lacking long-term risk estimates over 10 years, particularly for older adult and frail populations [43-45]. Third, many prior studies have small sample sizes or single-center designs, limiting statistical power for infrequent outcomes such as CKD [46,47]. More importantly, inadequate adjustment for competing risks in some analyses may lead to overestimation of disease-specific outcomes [48,49]. These limitations highlight the urgent need for large-sample, long-term, multicenter studies using robust statistical frameworks.

### Strengths of This Study

Compared with previous studies, this work offers several methodological advantages. The follow-up duration is extended, and leveraging nearly 2 decades of clinical data, this study can delineate long-term trajectories of BPH outcomes, overcoming the constraints of short- to midterm studies. The study will also use a multicenter, large-scale cohort of patients across various disease stages and treatment pathways, enhancing representativeness and external validity, with a sample size estimation that ensures sufficient power to detect low-incidence outcomes. The outcome assessment is comprehensive—beyond conventional end points such as surgery and urinary retention, the study incorporates inguinal hernia, CKD, urothelial carcinoma, and all-cause mortality, providing a more holistic evaluation of disease burden, thereby yielding further insights into the pathogenesis and progression of BPH and enhancing the robustness of statistical evidence. The statistical methodology is rigorous—depending on outcome characteristics, both Cox proportional hazard and Fine-Gray competing risk models will be used, with multivariable adjustments to minimize confounding and maximize robustness. Finally, the study will use a predictive modeling framework, building on identified risk factors to construct visual predictive tools (eg, nomograms) with internal validation and cross-validation across cohorts and quantify predictive performance using C-statistics. This addresses the lack of reliable prognostic tools for BPH outcomes.

### Expected Contributions

This study is expected to generate several important insights. First, it may identify novel risk factors beyond age and prostate volume, including metabolic parameters, comorbidity profiles, and initial treatment strategies, thereby improving early risk stratification. Second, it will provide authoritative, large-scale estimates of cumulative incidence and median time to event for major outcomes in the Chinese population, offering a robust evidence base for prognosis and health care planning.

### Future Perspectives

While retrospective cohort studies inherently face challenges [50-54]—such as potential data incompleteness from historical records and inherent biases—these limitations point to clear directions for future advancement. Looking ahead, 2 key priorities will drive follow-up efforts.

The first is expanding and enhancing data quality. Future work will integrate multisource data (including community-based patient records, primary care data, and long-term follow-up supplements) to fill gaps in existing datasets, paired with advanced data cleaning techniques to reduce measurement bias. This expansion will improve data accuracy, completeness, and external validity, making findings more applicable to diverse populations beyond hospital-based cohorts.

The second priority is transitioning to prospective research. Building on this study’s theoretical framework and preliminary insights, a dedicated prospective cohort will be designed to collect real-time clinical data—capturing dynamic BPH symptom changes, treatment responses, and long-term outcomes. Notably, leveraging long-term follow-up, multicenter design, and rigorous methodology, this prospective effort is expected to achieve significant breakthroughs in the study of BPH’s natural history and prognosis. Its results are poised to provide high-quality evidence-based support for optimizing patient management, implementing precision interventions, and formulating health care policies.

## Supplementary material

10.2196/84965 Multimedia Appendix 1 Univariable associations with time-to-event outcomes and multivariable models for events 1 to 6.

10.2196/84965 Checklist 1 STROBE checklist.

## Abbreviations

BPH: benign prostatic hyperplasia
CHARLS: China Health and Retirement Longitudinal Study
CKD: chronic kidney disease
EHR: electronic health record
LUTS: lower urinary tract symptoms
PLA: People's Liberation Army
STROBE: Strengthening the Reporting of Observational Studies in Epidemiology
