# Supplementary material for: Disease Progression and Outcomes in Patients With Benign Prostatic Hyperplasia: Protocol for a Multicenter Retrospective Cohort Study
Source: JMIR Res Protoc. 2026 Mar 2;15:e84965. doi: 10.2196/84965 (PMC12954690; doi:10.2196/84965)
Supplement: Multimedia Appendix 1 [file resprot-v15-e84965-s001.docx]

Table 3. Univariable Associations with Time-to-Event Outcomes.

Each cell reports effect size as HR/sHR (95% CI) and p-value from a univariable model. Events 1–5 are analyzed using Fine–Gray competing risk models (reporting sHR), and Event 6 is analyzed using Cox proportional hazards model (reporting HR).

| Variable | Event 1: | | Event 2: | | Event 3: | | Event 4: | | Event 5: | | Event 6: | |
| --- | --- | --- | --- | --- | --- | --- | --- | --- | --- | --- | --- | --- |
|  | sHR (95% CI) | p | sHR (95% CI) | p | sHR (95% CI) | p | sHR (95% CI) | p | sHR (95% CI) | p | HR (95% CI) | p |
| Residence |  |  |  |  |  |  |  |  |  |  |  |  |
| Age (years) |  |  |  |  |  |  |  |  |  |  |  |  |
| Body Mass Index (BMI, kg/m²) |  |  |  |  |  |  |  |  |  |  |  |  |
| Smoking status (Yes/No) |  |  |  |  |  |  |  |  |  |  |  |  |
| Alcohol consumption (Yes/No) |  |  |  |  |  |  |  |  |  |  |  |  |
| Hypertension (Yes/No) |  |  |  |  |  |  |  |  |  |  |  |  |
| Diabetes mellitus (Yes/No) |  |  |  |  |  |  |  |  |  |  |  |  |
| Coronary artery disease (Yes/No) |  |  |  |  |  |  |  |  |  |  |  |  |
| Psychiatric disorders (Yes/No) |  |  |  |  |  |  |  |  |  |  |  |  |
| Sleep disorders (Yes/No) |  |  |  |  |  |  |  |  |  |  |  |  |
| Respiratory diseases (Yes/No) |  |  |  |  |  |  |  |  |  |  |  |  |
| Constipation (Yes/No) |  |  |  |  |  |  |  |  |  |  |  |  |
| Medication Use |  |  |  |  |  |  |  |  |  |  |  |  |
| Serum creatinine (μmol/L) |  |  |  |  |  |  |  |  |  |  |  |  |
| Serum uric acid (μmol/L) |  |  |  |  |  |  |  |  |  |  |  |  |
| Triglyceride-to-HDL cholesterol ratio (TG/HDL) |  |  |  |  |  |  |  |  |  |  |  |  |
| Neutrophil-to-lymphocyte ratio (NLR) |  |  |  |  |  |  |  |  |  |  |  |  |
| Alanine aminotransferase (ALT, U/L) |  |  |  |  |  |  |  |  |  |  |  |  |
| D-dimer (mg/L) |  |  |  |  |  |  |  |  |  |  |  |  |
| Total PSA (ng/mL) |  |  |  |  |  |  |  |  |  |  |  |  |
| Free PSA (ng/mL) |  |  |  |  |  |  |  |  |  |  |  |  |
| Prostate volume (mL) |  |  |  |  |  |  |  |  |  |  |  |  |

Abbreviations: HR, hazard ratio; sHR, subdistribution hazard ratio; CI, confidence interval; PSA, prostate-specific antigen. Events were defined as follows: Event 1, surgical intervention for BPH; Event 2, urinary retention; Event 3, Inguinal hernia 4, CKD(**C**hronic **K**idney **D**isease) progression; Event 5, urothelial carcinoma; and Event 6, all-cause mortality.

Table 4. Multivariable models for events 1–3.

This table reports adjusted hazard ratios (aHR/sHR) with 95% CI. Events 1–5: Fine–Gray competing risk models (aSHR). Event 6: Cox proportional hazards model (aHR). Each outcome includes Models 1–3 (nested) and Model 4 (final).

| Variable | Event 1 | Event 1 | Event 1 | Event 1 | Event 2 | Event 2 | Event 2 | Event 2 | Event 3 | Event 3 | Event 3 | Event 3 |
| --- | --- | --- | --- | --- | --- | --- | --- | --- | --- | --- | --- | --- |
|  | M1 aSHR (95% CI) | M2 aSHR (95% CI) | M3 aSHR (95% CI) | M4 aSHR (95% CI) | M1 aSHR (95% CI) | M2 aSHR (95% CI) | M3 aSHR (95% CI) | M4 aSHR (95% CI) | M1 aSHR (95% CI) | M2 aSHR (95% CI) | M3 aSHR (95% CI) | M4 aSHR (95% CI) |
| Age (years) |  |  |  |  |  |  |  |  |  |  |  |  |
| BMI (kg/m²) |  |  |  |  |  |  |  |  |  |  |  |  |
| Hypertension (Yes/No) |  |  |  |  |  |  |  |  |  |  |  |  |
| … |  |  |  |  |  |  |  |  |  |  |  |  |

Table 5. Multivariable models for events 4–6.

| Variable | Event 4 | Event 4 | Event 4 | Event 4 | Event 5 | Event 5 | Event 5 | Event 5 | Event 6 | Event 6 | Event 6 | Event 6 |
| --- | --- | --- | --- | --- | --- | --- | --- | --- | --- | --- | --- | --- |
|  | M1 aSHR (95% CI) | M2 aSHR (95% CI) | M3 aSHR (95% CI) | M4 aSHR (95% CI) | M1 aSHR (95% CI) | M2 aSHR (95% CI) | M3 aSHR (95% CI) | M4 aSHR (95% CI) | M1 aHR (95% CI) | M2 aHR (95% CI) | M3 aHR (95% CI) | M4 aHR (95% CI) |
| Age (years) |  |  |  |  |  |  |  |  |  |  |  |  |
| BMI (kg/m²) |  |  |  |  |  |  |  |  |  |  |  |  |
| Hypertension (Yes/No) |  |  |  |  |  |  |  |  |  |  |  |  |
| … |  |  |  |  |  |  |  |  |  |  |  |  |

Abbreviations: aHR, adjusted hazard ratio; aSHR, adjusted subdistribution hazard ratio; CI, confidence interval; CKD, chronic kidney disease; BMI, body mass index. Events were defined as follows: Event 1, surgical intervention for BPH; Event 2, urinary retention; Event 3, Inguinal hernia ; Event 4, CKD progression; Event 5, urothelial carcinoma; and Event 6, all-cause mortality.
