# Supplementary material for: Disease Progression and Outcomes in Patients With Benign Prostatic Hyperplasia: Protocol for a Multicenter Retrospective Cohort Study
Source: JMIR Res Protoc. 2026 Mar 2;15:e84965. doi: 10.2196/84965 (PMC12954690; doi:10.2196/84965)
Supplement: Checklist 1 [file resprot-v15-e84965-s002.pdf]

STROBE Statement—Checklist of items that should be included in reports of ***cohort studies***

	Line No.	Recommendation
**Title and abstract**	1–3<br>22-68	(*a*) Indicate the study's design with a commonly used term in the title or the abstract
		(*b*) Provide in the abstract an informative and balanced summary of what was done and what was found
Background/rationale	100–146	Explain the scientific background and rationale for the investigation being reported
Objectives	147–151	State specific objectives, including any prespecified hypotheses
Study design	154–161	Present key elements of study design early in the paper
Setting	154–174	Describe the setting, locations, and relevant dates, including periods of recruitment, exposure, follow-up, and data collection
Participants	186–205	(*a*) Give the eligibility criteria, and the sources and methods of selection of participants. Describe methods of follow-up
	N/A	(*b*) For matched studies, give matching criteria and number of exposed and unexposed
Variables	162–166; 181–185; 208–216	Clearly define all outcomes, exposures, predictors, potential confounders, and effect modifiers. Give diagnostic criteria, if applicable
Data sources/ measurement	162–167; 206–207	For each variable of interest, give sources of data and details of methods of assessment (measurement). Describe comparability of assessment methods if there is more than one group
Bias	202–205; 252–263; 296–305	Describe any efforts to address potential sources of bias
Study size	246–263	Explain how the study size was arrived at
Quantitative variables	224–226; 306–307	Explain how quantitative variables were handled in the analyses. If applicable, describe which groupings were chosen and why
Statistical methods	264–285	(*a*) Describe all statistical methods, including those used to control for confounding
	N/A	(*b*) Describe any methods used to examine subgroups and interactions
	296-305	(*c*) Explain how missing data were addressed
	175-180;277-285	(*d*) If applicable, explain how loss to follow-up was addressed
	252-263;302-305	(*e*) Describe any sensitivity analyses
Results		
Participants	N/A (protocol)	(a) Report numbers of individuals at each stage of study—eg numbers potentially eligible, examined for eligibility, confirmed eligible, included in the study, completing follow-up, and analysed
		(b) Give reasons for non-participation at each stage
		(c) Consider use of a flow diagram
Descriptive data	N/A (protocol)	(a) Give characteristics of study participants (eg demographic, clinical, social) and information on exposures and potential confounders
		(b) Indicate number of participants with missing data for each variable of interest
		(c) Summarise follow-up time (eg, average and total amount)
Outcome data	N/A (protocol)	Report numbers of outcome events or summary measures over time

Main results	N/A (protocol)	(*a*) Give unadjusted estimates and, if applicable, confounder-adjusted estimates and their precision (eg, 95% confidence interval). Make clear which confounders were adjusted for and why they were included
		(*b*) Report category boundaries when continuous variables were categorized
		(*c*) If relevant, consider translating estimates of relative risk into absolute risk for a meaningful time period
Other analyses	N/A (protocol)	Report other analyses done—eg analyses of subgroups and interactions, and sensitivity analyses
**Discussion**		
Key results	407–410; 446–451	Summarise key results with reference to study objectives
Limitations	411–423	Discuss limitations of the study, taking into account sources of potential bias or imprecision. Discuss both direction and magnitude of any potential bias
Interpretation	424–444; 453–471	Give a cautious overall interpretation of results considering objectives, limitations, multiplicity of analyses, results from similar studies, and other relevant evidence
Generalisability	168–174; 333–338; 430–432	Discuss the generalisability (external validity) of the study results
**Other information-**		
Funding	356–366; 472–480	Give the source of funding and the role of the funders for the present study and, if applicable, for the original study on which the present article is based

*Give information separately for exposed and unexposed groups.
